# *Sargassum mcclurei* Mitigating Methane Emissions and Affecting Rumen Microbial Community in In Vitro Rumen Fermentation

**DOI:** 10.3390/ani14142057

**Published:** 2024-07-13

**Authors:** Shuai Li, Yi Sun, Tongjun Guo, Wenyou Liu, Xiong Tong, Zhifei Zhang, Jiajie Sun, Yufeng Yang, Shuli Yang, Dagang Li, Li Min

**Affiliations:** 1 Southern Marine Science and Engineering Guangdong Laboratory (Zhuhai), Ministry of Agriculture Key Laboratory of Animal Nutrition and Feed Science in South China, Institute of Animal Science, Guangdong Academy of Agricultural Sciences, Guangzhou 510640, China; ls32649048@163.com (S.L.); sunyi@stu2022.jnu.edu.cn (Y.S.); lwy5205202023@163.com (W.L.); tongxiong@gdaas.cn (X.T.); zhangzhifei@gdaas.cn (Z.Z.); 2 Guangdong Provincial Key Laboratory of Animal Nutrition Regulation, College of Animal Science, South China Agricultural University, Guangzhou 510642, China; jiajiesun@scau.edu.cn; 3 Southern Marine Science and Engineering Guangdong Laboratory (Zhuhai), College of Life Science and Technology, Jinan University, Guangzhou 510632, China; tyyf@jnu.edu.cn; 4 Key Laboratory of Xinjiang feed biotechnology, Feed Research Institute, Xinjiang Academy of Animal Science, Urumqi 830000, China; guotaoxj@126.com; 5 College of Life Sciences and Engineering, Foshan University, Foshan 528231, China; yangshuli77@163.com

**Keywords:** rumen fermentation, seaweed, methane emissions, rumen microbiota

## Abstract

**Simple Summary:**

Methane (CH_4_) is a by-product of ruminant microbial fermentation, leading to a 2–12% loss of feed energy. The greenhouse gas effect of methane in the atmosphere is 28 times greater than that of carbon dioxide. Recent studies have shown that supplementing feed additives can effectively reduce ruminal methane emissions. This study aimed to evaluate the effectiveness of *Sargassum mcclurei* (*S. mcclurei*) in mitigating methane emissions using different treatment methods and supplementation levels through in vitro rumen fermentation. Three supplementation levels were tested for both dried and freeze-dried treatments over 48 h of in vitro rumen fermentation. Fermentation gas production was recorded, and after fermentation, methane production, dry matter degradation (DMD), and various fermentation parameters were measured. The addition of *S. mcclurei* affected crude protein degradation (CPD) and volatile fatty acid (VFA) production. The freeze-dried *S. mcclurei* at a 2% supplementation level reduced methane emissions by 18.85%.

**Abstract:**

Methane emissions from ruminants significantly contribute to greenhouse gases. This study explores the methane mitigation effect and mechanism of *S. mcclurei* through in vitro rumen fermentation, aiming to establish its potential as a feed additive. We investigated the effects of freeze-dried and dried *S. mcclurei* at supplementation levels of 2%, 5%, and 10% of dry matter on nutrient degradation, ruminal fermentation, methane inhibition, and microbial community structure in in vitro rumen fermentation. The freeze-dried *S. mcclurei* at 2% supplementation significantly reduced CH_4_ emissions by 18.85% and enhanced crude protein degradability. However, total VFA and acetate concentrations were lower in both treatments compared to the control. The microbial shifts included a decrease in *Lachnospiraceae_NK3A20_group* and *Ruminococcus* and an increase in *Selenomonas*, *Succinivibrio*, and *Saccharofermentans*, promoting propionate production. Additionally, a significant reduction in *Methanomicrobium* was observed, indicating direct methane mitigation. Freeze-dried *S. mcclurei* at a 2% supplementation level shows potential as an effective methane mitigation strategy with minimal impact on rumen fermentation, supported by detailed insights into microbial community changes.

## 1. Introduction

Greenhouse gas (GHG) emissions lead to global warming. Livestock emissions of GHGs, a significant source of the agricultural sector, account for about 15% of annual anthropogenic emissions [1]. Ruminants, particularly beef and dairy cattle, contribute most to GHG emissions from livestock [1]. Methane emissions from enteric fermentation represent about 41% of total GHG emissions from the agricultural sector [2]. It is widely recognized that the mitigation of CH_4_ emissions from ruminants is crucial to address GHG emissions. Various feed additives have demonstrated the potential to mitigate ruminal CH_4_ emissions by regulating pathways involved in microbial methanogenesis [3,4]. Of these, seaweed has been shown as a promising feed additive for mitigating rumen CH_4_ emissions. Seaweeds are among the world’s largest unexploited and renewable global biomass resources, with the total production of macroalgae reaching 32.4 million tons in 2018 across approximately 50 countries [5]. Asia, particularly China, has seaweed production contributing over 97% of the world’s production with more than 18 million tons [6].

Seaweeds contain many bioactive compounds, two of which are particularly noteworthy: bromoform in red seaweed and phlorotannins in brown seaweed. Both of these compounds are known as CH_4_ inhibitors [7,8]. However, the bioactive components of seaweed are affected by many factors, such as species, season, and the location of harvesting [9]. Current studies have indicated that terrestrial tannins can effectively mitigate CH_4_ production [10,11]. It has been reported that phlorotannins have chemical structural similarities to terrestrial tannins [12]. The *S. mcclurei* used in our study is a brown seaweed rich in phlorotannins. Therefore, we hypothesize that phlorotannins in brown seaweed may have a CH_4_-mitigating effect similar to that of terrestrial tannins. However, the effectiveness of reducing methane emissions is related to seaweed supplementation levels [7,8] and also processing methods including freeze-drying and drying [4,13]. According to reports, freeze-drying can extend the storage time of biomass in seaweed, which might play a vital role in CH_4_ emission [14].

Hence, this study aims to explore the effects of the in vitro rumen fermentation of brown seaweed, *S. mcclurei*, with two treatments (freeze-drying and drying) and three supplementation levels (0, 2, 5, and 10% of DM basis) on nutrient degradation, rumen fermentation parameters, methane emission inhibition effect, and microbial community structure, thereby evaluating the use of *S. mcclurei* as a feed additive to feasibly alleviate rumen greenhouse gas emissions.

## 2. Materials and Methods

### 2.1. Preparation of Seaweed and TMR Substrate

The brown seaweed of *S. mcclurei* was harvested from Weizhou Island (Beihai, Guangxi, China). The seaweed was submerged for 1 min in freshwater and divided into two equal portions. Each portion was then dried through a spin-dryer until no water continued to flow out. One portion was frozen at −80 °C for 24 h then vacuum freeze-dried at −30 °C and 0.37 vacuum degrees for 48 h (CHRIST ALPHA2-4, Osterode, Germany). The second portion was immediately dried in an oven at 65 °C for 48 h (DHG-9075A, Shanghai, China). The samples of seaweed were milled to 1 mm and stored at −20 °C. The Total Mixed Ration (TMR) that constituted corn straw and concentrate supplementation was milled to 1 mm and stored in a silica gel desiccator.

Dry matter (DM) was determined by the achievement of constant weight at 105 °C, and organic matter (OM) was measured as the loss on combustion at 550 °C for 8 h [15]. Neutral and acid detergent fiber (NDF and ADF) content were analyzed following the methods outlined by Van Soest [16] and crude protein (CP) was analyzed by the method of Thiex [17].

### 2.2. In Vitro Treatment

Three healthy Holstein cows were selected as rumen fluid donors and fed twice a day at 7:00 and 17:00 with free drinking water. Rumen fluid was collected 2 h post-morning feeding, blended, and filtered separately using a four-layer cheesecloth into a pre-warmed thermos flushed with carbon dioxide (CO_2_) before collection. The rumen fluid was rapidly transferred to the laboratory and mixed with rumen fluid buffer solution at a ratio of 1:2 (rumen fluid: buffer solution), as in the method of Menke [18], and maintained in water at 39 °C, ensuring continuous CO_2._

The mixed rumen fluid (75 mL) was dispensed into 100 mL incubation bottles containing 500 mg of a TMR substrate (60:40 corn straw: concentrate supplementation) and two different treatments of *S. mcclurei* (freeze-dried and dried). Four levels of supplementation (0, 2, 5, and 10% of DM basis) were set for each treatment. Six biological replicates were set for each supplementation level, and the experiment was repeated twice. Six samples were randomly selected for subsequent experimental analysis. These varying supplementation levels were designed to determine the optimal level for mitigating CH_4_ emissions and to evaluate the potential adverse effects of high supplementation levels on rumen fermentation [13].

The incubation bottles were rinsed with CO_2_, sealed with butyl plugs and aluminum caps, and placed into a constant temperature water bath shaker at 39 °C and 85 RPM for 48 h.

### 2.3. Experimental Sample Collection and Analysis

#### 2.3.1. Gas Production Collection

The total gas production was collected from the incubation bottles at 2, 4, 8, 12, 24, and 48 h using a 30 mL syringe. The samples were stored in aluminum foil airbags until analysis. Concentrations of CH_4_ and CO_2_ were analyzed by gas chromatography with a 5A ZSF-SS column (Φ3 mm × 3 m, Support 60–80 mesh Chromosorb) and a thermal conductivity detector (SP-2060T, Beijing, China). The detector is at a constant temperature, and Argon (Ar) as the carrier gas has a flow rate of 30 mL/min.

#### 2.3.2. Fermentation Parameter Determination

At the end of the treatment (48 h), the fermenter pH was immediately measured. Three 5 mL rumen fluid samples were placed in centrifuge tubes for analysis of VFA composition, microprotein (MCP), and ammonia nitrogen (NH_3_-N) concentrations, respectively. Samples were stored at −80 °C until analysis. The concentration of VFA was analyzed using gas chromatography (Agilent 6890N, Palo alto, CA, USA) as described by [19]. The determination of NH_3_-N and MCP was conducted as described by Vissers [20]. The in vitro rumen fermentation of nutrient digestibility was measured using the substrate collected in a nylon bag after incubation. The bags were washed with fresh water until clear, then over-dried at 65 °C to a constant weight. Briefly, the digestibility of DM, CP, NDF, and ADF (DMD, CPD, NDFD, and ADFD) was defined as weight loss compared to the pre-incubation weights in the incubation bottles.

#### 2.3.3. DNA Extraction and Bacterial and Archaeal 16S rRNA Gene Sequencing and Analysis

Total genomic DNA was extracted from in vitro fermentation rumen fluid samples using the E.Z.N.A.^®^ soil DNA Kit (Omega Bio-tek, Norcross, GA, USA) following the manufacturer’s instructions. The hypervariable region V3-V4 of the bacterial 16S rRNA gene was amplified with primer pairs 338F (5′-ACTCCTACGGGAGGCAGCAG-3′) and 806R (5′-GGACTACHVGGGTWTCTAAT-3′) by a T100 Thermal Cycler PCR thermocycler (BIO-RAD, Hercules, CA, USA). The e V4-V5 region of the archaeal 16S rRNA genes was amplified by 349F (5′-GYGCASCAGKCGMGAAW-3′) and 806R (5′-GGACTACVSGGGTATCTAAT-3′). The PCR reaction mixture included 10 ng of template DNA, 0.8 μL of each primer (5 μM), 2 μL of 2.5 mM dNTPs, 0.4 μL of Fast Pfu polymerase, 4 μL of 5 × Fast Pfu buffer, and ddH2O to a final volume of 20 µL. PCR amplification cycling conditions were as follows: initial denaturation at 95 °C for 3 min, followed by 27 cycles of denaturing at 95 °C for 30 s, annealing at 55 °C for 30 s, extension at 72 °C for 45 s, single extension at 72 °C for 10 min, and ending at 4 °C. The PCR product was extracted from 2% agarose gel and purified using the PCR Clean-Up Kit (YuHua, Shanghai, China) according to the manufacturer’s instructions and quantified using Qubit 4.0 (Thermo Fisher Scientific, Waltham, MA, USA).

Bioinformatic analysis of the rumen microbiota was carried out using the Majorbio Cloud platform (https://cloud.majorbio.com, accessed on 24 November 2023). Raw FASTQ files were de-multiplexed using an in-house perl script and then quality-filtered by fast version 0.19.6 and merged by FLASH version 1.2.7. Next, the primers and barcodes were removed, and chimeras were filtered to obtain valid reads. After filtration, the numbers of valid reads of bacterial and archaeal communities were 1,117,522 and 1,494,549, respectively, and the average lengths of valid reads were 419 and 375, respectively. Then, the optimized sequences were clustered into operational taxonomic units (OTUs) using UPARSE 7.1 with a 97% sequence similarity level. The most abundant sequence for each OTU was selected as a representative sequence. To minimize the effects of sequencing depth on alpha and beta diversity measure, the number of 16S rRNA gene sequences from each sample was rarefied to 20,000, which still yielded an average Good’s coverage of 99.09%.

Based on the OTU information, rarefaction curves and alphas were calculated with Mothur v1.30.1. The similarity among the microbial communities in different samples was determined by principal coordinate analysis (PCoA) based on Bray–Curtis dissimilarity using the Vegan v2.5-3 package. The linear discriminant analysis (LDA) effect size (LEfSe) (http://huttenhower.sph.harvard.edu/LEfSe, accessed on 24 November 2023) was performed to identify the significantly abundant taxa (phylum to genera) of bacteria among the different groups (LDA score > 2, *p* < 0.05).

### 2.4. Statistical Analysis

One-factor ANOVA was used to test for significant differences in the fermenter parameter (pH, DMD, NDFD, and ADFD), the total gas produced over time, and the production of CH_4_ and CO_2_ during the experimental period. A one-factor ANOVA was also used to test for significant differences in VFA, MCP, and NH_3_-N concentration. All ANOVA analyses were performed using IBM SPSS (27.0) software. *p* < 0.05 was considered a significant difference.

## 3. Results

### 3.1. Effects of S. mcclurei on the CH_4_ Production and Nutrient Degradation of In Vitro Rumen Fermentation

As shown in Table 1, the total gas production (mL/g DM) was decreased (*p* < 0.05) in all treatment groups compared to control group (CON) during the incubation period. The decrease was affected by the supplementation of *S. mcclurei*, with the lowest yields observed at the 10% inclusion level for both the dried and freeze-dried treatments. The total gas production decreased by 13.96% (*p* < 0.01) and 11.61% (*p* = 0.02), respectively. Each supplementation level in the dried group had the effect of mitigating CH_4_ emissions, but the difference in mitigation effect between each supplementation level was not significant. Furthermore, the 2% supplementation level in the freeze-dried group had the most effective mitigation of CH_4_ emission (18.85%, *p* < 0.05), while the other two supplementation levels had no significant effect on CH_4_ concentration. No significant difference in CO_2_ production was observed between CON and treatment groups.

The chemical composition of the diet and *S. mcclurei* in the study is shown in Table 2. Different treatments and supplementation levels of *S. mcclurei* influenced the degradation of all analyzed nutrients in the incubation bottles (Table 3). Notably, nutrient degradation (DMD, NDFD, and ADFD) tended to be inhibited in all treatment groups when the seaweed supplementation level was 10%, compared to the CON. In the freeze-dried group, the degradation of crude protein increased with the supplementation of low levels (2% of substrate DM) of *S. mcclurei*. However, as the supplementation level increased, the degradation of crude protein was inhibited.

### 3.2. Effects of S. mcclurei on the Fermentation Characteristics of In Vitro Rumen Fermentation

The effects of the seaweed on the rumen fermentation characteristics are described in Table 4. The pH and NH_3_-N were not affected by any of the supplemented treatments after 48 h of incubation. However, the drying treatment did increase the concentration of MCP (*p* < 0.05), with the MCP concentration increasing linearly with the level of supplementation.

Supplementation with both the dried treatment (*p* < 0.01) and freeze-dried treatment (*p* = 0.02) of *S. mcclurei* significantly reduced total VFA concentrations in the in vitro rumen fermentation compared to the CON. The decrease in total VFA concentration was more pronounced in the dried group. The supplementation of two different treatments of *S. mcclurei* significantly reduced the concentrations of total VFA, acetate (*p* < 0.01), and butyrate (*p* < 0.01) in the in vitro rumen fermentation. The highest supplementation level in the dried group corresponded to the lowest total VFA, acetate, and butyrate concentrations. Conversely, the lowest total VFA, acetate (*p* < 0.01), and butyrate (*p* < 0.01) concentrations in the freeze-dried group were observed at the lowest supplementation level. The propionate concentrations in the two treatment groups showed no significant difference from those in the CON. Both the dried group (*p* < 0.01) and the 2% supplementation level in the freeze-dried group (*p* < 0.01) significantly decreased the ratio of acetate/propionate.

### 3.3. Changes in the Microbial Composition

#### 3.3.1. Effects of Different Treatments on Bacterial Community at the 2% Supplementation Level

Based on the above experimental results of gas production, nutrient degradation, and fermentation characteristics, we presume that the 2% supplementation level is the best solution for seaweed addition in this trial. We further analyzed the microbial communities at the 2% supplementation level in both treatment groups and CON, and compared the effects of different treatments of *S. mcclurei* supplementation on rumen microorganisms at the same level.

As shown in Table 5, the alpha diversity of the bacterial community, observed abundance-based coverage estimator (ACE), Chao 1, Shannon, and Simpson indices did not significantly differ among different treatments (*p* > 0.05). This result shows that the supplementation of *S. mcclurei*, whether freeze-dried or dried, did not affect the diversity and richness of the bacterial community.

**Figure 1:**
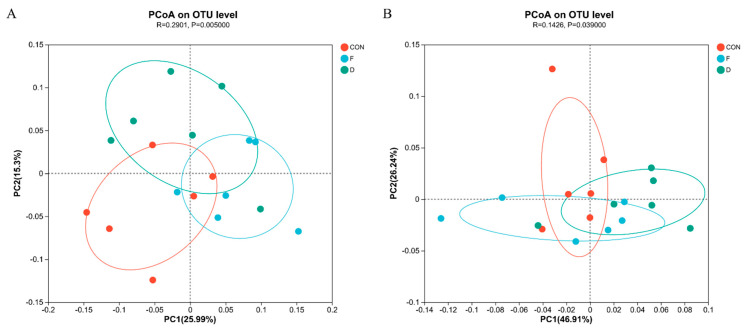
Principal coordinate analysis (PCoA) based on Bray–Curtis distance of the rumen bacterial (**A**) and archaeal (**B**) communities in in vitro rumen fermentation. CON, control group; F, freeze-dried treatment (2% of substrate DM); D, dried treatment (2% of substrate DM).

The bacterial community beta diversity with different treatments was analyzed by principal coordinate analysis (PCoA) based on the Bray–Curtis distance, as shown in Figure 1A. ANOSIM revealed a significant difference in the bacterial community composition (R = 0.2901, *p* = 0.005).

**Figure 2:**
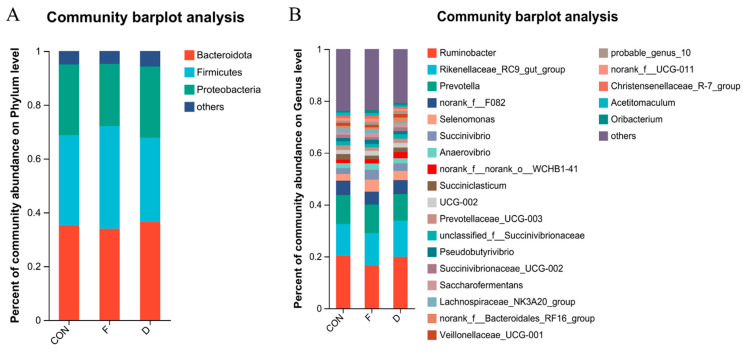
The rumen bacterial community composition in the CON, freeze-dried treatment, and dried treatment groups. (**A**) Phylum level; (**B**) genus level. CON, control group; F, freeze-dried treatment (2% of substrate DM); D, dried treatment (2% of substrate DM).

In this trial, at the phylum level, Bacteroidota, Firmicutes, and Proteobacteria were the dominant phyla (Figure 2A). At the genus level, 23 genera had a relative abundance of >0.1%. Twenty genera with a relative abundance of >0.1% were identified: *Ruminobacter*, *Rikenellaceae_RC9_gut_group*, *Prevotella, norank_f__F082*, *Selenomonas*, *Succinivibrio*, *Anaerovibri*, *norank_f__norank_o__WCHB1-41*, *Succiniclasticum*, UCG-002, *Prevotellaceae_UCG-003*, *unclassified_f__Succinivibrionaceae*, *Pseudobutyrivibrio*, *Succinivibrionaceae_UCG-002*, *Saccharofermentans*, *Lachnospiraceae_NK3A20_group*, *norank_f__Bacteroidales_RF16_group*, *Veillonellaceae_UCG-001*, probable_genus_10, *norank_f__UCG-011*, *Christensenellaceae_R-7_group*, *Acetitomaculum*, and *Oribacterium*, as shown in Figure 2B.

**Figure 3:**
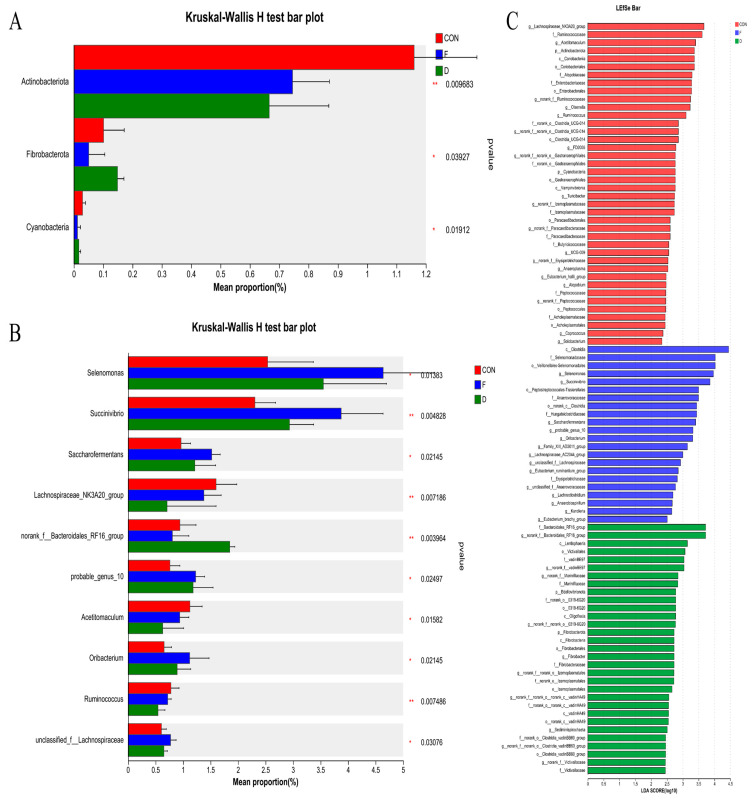
Principal differences in the relative abundances of bacterial phyla and histograms with LDA scores > 2 were calculated for each taxonomic unit from phylum to genus. (**A**) Phylum level; (**B**) genus level; (**C**) the LDA values for different differential species. CON, control group; F, freeze-dried treatment (2% of substrate DM); D, dried treatment (2% of substrate DM).

At the phylum level, the supplementation of *S. mcclurei* after freeze-drying treatment decreased the relative abundance of Actinobacteriota (*p* < 0.05), Fibrobacterota (*p* < 0.05), and Cyanobacteria (*p* < 0.01) in the in vitro rumen fermentation. Supplementing dried *S. mcclurei* increased the relative abundance of Fibrobacterota (*p* < 0.05) and decreased the abundance of Actinobacteriota (*p* < 0.05) and Cyanobacteria (*p* < 0.01, Figure 3A). At the genus level, the results of this experiment showed that the supplementation of *S. mcclurei* after both treatments increased the abundance of *Selenomonas* (*p* < 0.05), *Succinivibrio* (*p* < 0.01), *Saccharofermentans* (*p* < 0.05), *probable_genus_10* (*p* < 0.05), *Oribacterium* (*p* < 0.05), and *unclassified_f_Lachnospiraceae* (*p* < 0.05). Furthermore, *S. mcclurei* after drying treatment supplementation significantly increased the abundance of *norank_f__Bacteroidales_RF16_group* (*p* < 0.01), and the abundance of *Lachnospiraceae_NK3A20_group* (*p* < 0.01), *Acetitomaculum* (*p* < 0.05), and *Ruminococcus* (*p* < 0.01) were significantly decreased (Figure 3B). LEfSe analysis was performed using all microbial data to determine key bacterial groups associated with the freeze-drying and drying treatments. Figure 3C depicts the significantly different bacteria between the CON and the two treatment groups. The most significant bacterial genera in the CON were *Lachnospiraceae_NK3A20_group* and *Acetitomaculum*, and the bacterial genera with the most difference in the freeze-dried and dried groups were *Selenomonas*, *Succinivibrio*, and *RF16*, respectively.

#### 3.3.2. Effects of Different Treatments on the Archaeal Community at the 2% Supplementation Level

**Figure 4:**
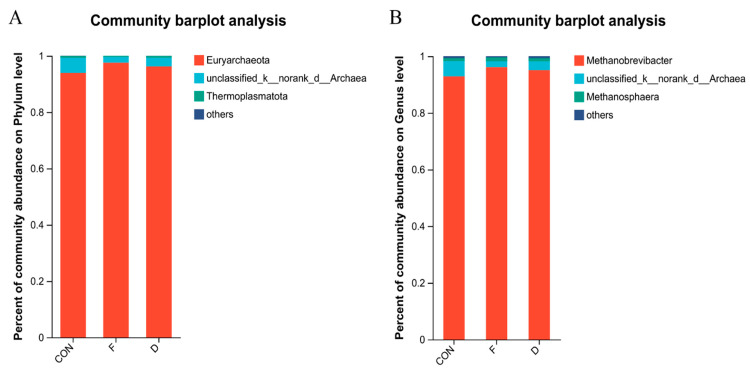
The rumen archaeal community composition in CON, freeze-dried treatment, and dried treatment groups. (**A**) Phylum level; (**B**) genus level. CON, control group; F, freeze-dried treatment (2% of substrate DM); D, dried treatment (2% of substrate DM).

**Figure 5:**
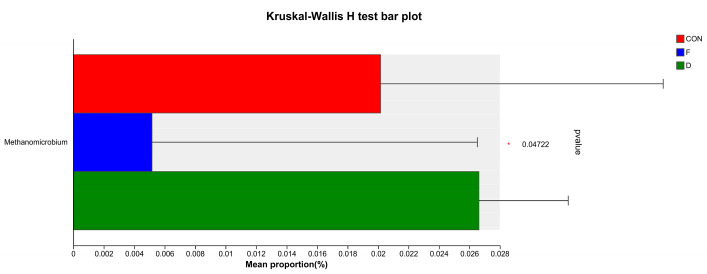
Principal differences in the relative abundances of the archaeal genera. CON, control group; F, freeze-dried treatment (2% of substrate DM); D, dried treatment (2% of substrate DM).

The alpha diversity of the archaeal community is detailed in Table 5, revealing no significant differences in ACE, Chao 1, Shannon, or Simpson indices among all treatment groups (*p* > 0.05). This suggests that the diversity and richness of the archaeal community were not affected by *S. mcclurei* supplementation with different treatments. The beta-diversity analysis of the archaea community is consistent with the above-mentioned bacteria and the results are depicted in Figure 1B. At the archaeal phylum level, Euryarchaeota was the most dominant phylum (Figure 4A). At the genus level, *Methanobrevibacter* was the most dominant genera (Figure 4B). The supplementation of dried or freeze-dried *S. mcclurei* had no significant difference on the archaeal community at the phylum level. At the genus level, the freeze-dried group significantly reduced the abundance of *Methanomicrobium* (*p* < 0.05), and there were no significant differences between CON and the dried group (Figure 5).

## 4. Discussion

The supplementation of seaweed in in vitro rumen fermentation has been reported to affect nutrient degradation, fermentation characteristics, and microbial composition [4,21,22]. In the current study, supplementation with *S. mcclurei*, a kind of brown seaweed, did not affect DMD and NDFD but lowered the CPD in the freeze-dried group when the seaweed supplementation level was 10% (Table 3). Meanwhile, a decreasing trend of ADFD was observed in the freeze-dried group as the supplementation increased, but the difference was not significant (Table 3). The results suggest that high supplementation levels also harm the degradation of ruminal fermentation nutrients. However, the 2% supplementation level has no negative impact on the degradation of nutrients, except for the degradation of crude protein. Based on the results, we speculate that a 2% *S. mcclurei* supplementation level is more suitable than a higher supplementation level in this experiment. Currently, there is limited research on the effects of *S. mcclurei* on nutrient degradation. This experiment will improve the information in this field, thereby guiding the production application.

This study explores the effects of *S. mcclurei* supplementation on the rumen fermentation parameters, providing insight into the complex dynamics of the rumen environment. The fermentation parameters of pH, VFA, NH_3_-N, and MCP reflect the stability of the internal rumen environment [23]. In this study, pH and NH_3_-N concentrations showed no effect. Higher MCP concentration and lower VFA concentration were observed as a result of supplementation with *S. mcclurei* (Table 4). *S. mcclurei* contains 12% high-quality protein and when added to rumen may be utilized by rumen microorganisms to produce more MCP. The results show that, in addition to reducing VFA concentration, *S. mcclurei* supplementation has no other negative effects on rumen fermentation parameters and internal environment stability.

The dried group significantly reduced the total VFA, acetate, and butyrate concentrations, while the total VFA, acetate, and butyrate also decreased significantly when the freeze-dried group was added at the 2% level (Table 4). This observation suggests that different treatments and supplementation levels of *S. mcclurei* alter the pattern of VFA production by fermentation in the rumen. The inhibitory effect of brown seaweed on VFA concentration has also been reported. For example, Kunzel [8] observed that the supplementation of *Ascophyllum nodosum* and *Fucus vesiculosus* decreased the total VFA concentration. Beauchemin [24] reported that rumen microorganisms ferment lignocellulose through the acetate pathway, releasing H_2_ and causing higher CH_4_ concentrations. Simultaneously, propionic production is thought to be the major H_2_ sink in the rumen when CH_4_ production is inhibited [25]. Wettstein [26] also reported that decreased CH_4_ concentration sometimes shifts microbial fermentation from acetate to propionate. This shows that there may be a positive correlation between acetate and CH_4_, while propionate may be negatively correlated with CH_4_ in rumen fermentation [27]. In this experiment, the propionate concentration was not affected by the supplementation of *S. mcclurei*, but the acetate–propionate ratio (A:P) significantly decreased. This is more conducive to the mitigation of CH_4_ emissions, consistent with previous research results [13,28]. Subsequently, it is necessary to conduct further exploration of the specific relationship between VFA production and CH_4_ emissions in rumen fermentation.

In the current study, different processing treatments and supplementation levels significantly affected the CH_4_ concentration of in vitro rumen fermentation. It is worth noting that the freeze-dried treatment performed exceptionally well at a 2% supplementation level, decreasing CH_4_ concentration by 18.85% without negatively affecting nutrient degradation (Table 1). The results demonstrate the first evidence that a low-dose (2% of substrate DM) freeze-dried treatment of *S. mcclurei* can effectively reduce CH_4_ emissions compared to other brown seaweeds including *Sargassum*, *Cystoseira*, *Ascophyllum nodosum*, and *Fucus vesiculosus* [4,8,29]. As mentioned above, brown seaweed including *S. mcclurei*, inhibits CH_4_ production, primarily attributed to the presence of phlorotannins, which are compounds found richly in brown seaweed. The phlorotannins vary in concentration between species and have significant differences among the different brown seaweed species [30]. Therefore, we presume that the active ingredient in *S. mcclurei*, phlorotannins, might play an important role in the process of reducing methane emissions in this experiment. Comparatively, the freeze-dried treatment is more effective in preserving phlorotannins than the dried treatment, resulting in a higher phlorotannins content in the freeze-dried group at the same supplementation level [31,32]. This observed phenomenon prompts an exploration into the potential correlation between the concentration of phlorotannins and their mitigating effect on CH_4_ emission during in vitro rumen fermentation. However, there definitely exists an upper limit of the inhibitory effect on CH_4_ emissions by phlorotannins. A higher supplementation level of *S. mcclurei* could not further reduce the CH_4_ mitigation but might harm fermentation. This hypothesis agrees with the observed optimal performance at the 2% supplementation level in the freeze-dried treatment in our experiment, suggesting a balance between CH_4_ reduction and minimal negative impact on fermentation.

CH_4_ emissions from ruminants are intricately tied to the rumen microbiome, particularly focusing on methanogenic archaea that synthesize CH_4_ as the end product of anaerobic fermentation [33,34]. In this study, we elucidated the effects of dried and freeze-dried treatments at the 2% supplementation level on ruminal microbial composition and functions, contributing to verifying the effect of *S. mcclurei* on methanogenesis in rumen fermentation in vitro. Our findings revealed that both dried and freeze-dried seaweed supplementation did not influence the α-diversity indices (ACE, Chao 1, Shannon, Simpson). These indices, reflecting microbial diversity and richness, indicated that bacterial diversity and richness in the rumen fermentation in vitro were not altered (Table 5).

The dominant bacteria in the rumen microbial community were identified as Bacteroidota, Firmicutes, and Proteobacteria, similar to several other studies [35,36,37] (Figure 2A). At the phylum level, changes in the bacterial community structure were evident (Figure 3A). The relative abundance of Actinobacteriota and Cyanobacteria significantly decreased in all treatment groups compared with CON. Conversely, the relative abundance of Fibrobacterota exhibited a trend of increase. Actinobacteriota, Fibrobacterota, and Cyanobacteria had a significant impact on starch and cellulose degradation [38,39]. These observed changes in their abundance may contribute to the decrease in total VFA concentration. To further analyze the effect of different treatments at the 2% supplementation level on in vitro rumen fermentation, we analyzed the bacterial community structure at the genus level, as shown in Figure 2B.

Intriguingly, *Selenomonas*, *Succinivibrio*, and *Saccharofermentans’* relative abundance significantly increased in the freeze-dried group (Figure 3B). Xue [33] reported that *Selenomonas* is positively correlated with several genera of the family *Succinivibrionaceae*, including *Succinivibrio*. *Selenomonas* can ferment starch to produce lactate, acetate, and propionate, while *Succinivibrio* and *Saccharofermentans* are involved in utilizing fermentation products to produce succinate, lactate, acetate, and formate and transfer H_2_ away from methanogenesis [40]. The higher relative abundance of these genera in the freeze-dried group was associated with significant decreases in CH_4_ concentration compared to the CON. These genera were positively correlated with propionate concentration; however, there was no increase in propionate concentration in this study. We hypothesized that the relatively low abundance of *Selenomonas* (2.53–4.63%), *Succinivibrio* (2.30–3.87%), and *Saccharofermentans* (0.96–1.52%) might not have a significant effect on propionate production. The production of acetate bacteria such as *Lachnospiraceae_NK3A20_group*, *Ruminococcus*, and *Acetitomaculum* [41,42] exhibited a significant decrease in relative abundance in both treatment groups, with a more pronounced effect in the dried group (Figure 3B). These changes agree with a lower acetate concentration in both treatment groups compared to CON, with the dried group evidencing the lowest acetate concentration. The reduction in the relative abundance of these bacteria contributed to the observation of a decrease in acetate production. *Probable_genus_10*, for which the relative abundance significantly increased in both treatment groups, was also shown to promote propionate production [36]. Overall, *S. mcclurei* supplementation in both treatments changes the rumen fermentation pattern by altering the relative abundances of bacteria, which allows more H_2_ to be transferred to the pathway of propionate production, thereby mitigating CH_4_ emission. Compared with the two treatments, the freeze-dried treatment demonstrated a more pronounced effect. It produced more propionate (2.07%), more effectively mitigating the negative effect on acetate production (0.81%), and demonstrated superior efficacy in inhibiting CH_4_ emissions. This understanding of the intricate relationships between microbial communities, rumen fermentation, and CH_4_ production highlights the potential of freeze-dried *S. mcclurei* as a promising supplement for mitigating CH_4_ emissions in ruminant systems.

Archaea are widely present in the rumen and can utilize H_2_ to maintain the fermentation environment of rumen microorganisms and the production of CH_4_ [33]. In our trial, the phylum Euryarchaeota was the most dominant (Figure 4A), aligning with the findings of Liu [43]. Euryarchaeota is recognized as a classic methanogen in the rumen. Thaumarchaeota, identified in our study, are considered among the most abundant archaea globally. Both archaea species are thought to have an impact on climate change [44]. At the genus level (Figure 4B), *Methanobrevibacter*, *unclassified_k__norank_d__Archaea*, and *Methanosphaera* were identified. *Methanobrevibacter*, the predominant rumen methanogen, produces CH_4_ from CO_2_ via the hydrogenotrophic pathway and correlates positively with CH_4_ production [45]. Conversely, *Methanosphaera*, known for consuming methanol to produce CH_4_ through the methylotrophic pathway, has been associated with negative correlations with CH_4_ production [46]. Remarkably, the only notable change observed was in the relative abundance of *Methanomicrobium*, which significantly decreased in the freeze-dried group compared to CON (Figure 5). *Methanobrevibacter*, belonging to the hydrogenotrophic methanogens within the phylum Euryarchaeota, also exhibits positive correlations with CH_4_ production, deriving CH_4_ through the reduction of CO_2_ [45].

## 5. Conclusions

This study investigated the effect of supplementing freeze-dried and dried *S. mcclurei* on in vitro rumen fermentation, microbiota, and CH_4_ production using the same basal total mixed ration. The freeze-dried treatment at the 2% addition level increased the degradation rate of crude protein, had a positive effect on the rumen fermentation in vitro, and reduced CH_4_ emissions by 18.85%. The supplementation influenced the microbial composition by increasing the relative abundance of *Selenomonas*, *Succinivibrio*, and Saccharofermentans while decreasing the abundance of *Methanomicrobium*. The increases in the relative abundance of these bacteria change the ruminal fermentation pattern, allowing more hydrogen to be transferred to the propionate production pathway and competitively inhibiting CH_4_ production. The decreases in the relative abundance of *Methanomicrobium* directly reduce CH_4_ production in in vitro rumen fermentation. The findings of this study offer valuable insights into the potential of freeze-dried *S. mcclurei* supplementation as a strategy for CH_4_ reduction in ruminants and pave the way for practical application.

## Figures and Tables

**Table 1:** The cumulative total gas and gas composition (CH_4_ and CO_2_) of the two treatments and different supplementation levels after in vitro rumen fermentation for 48 h.

Parameter	CON	2%	5%	10%	*p*
D					
TGP mL	142.48 ± 15.22	138.43 ± 2.13	124.77 ± 14.7 9	134.86 ± 4.19	0.06
CH_4_ mL	12.08 ± 2.27	10.63 ± 0.78	10.11 ± 1.16	10.07 ± 0.77	0.07
CO_2_ mL	89.79 ± 21.28	90.80 ± 5.76	82.28 ± 9.10	93.27 ± 3.66	0.44
TGP mL/g DM	284.62 ± 30.35 ^A^	271.02 ± 4.17 ^A^	237.34 ± 28.18 ^B^	244.86 ± 7.59 ^B^	<0.01
CH_4_ mL/g DM	24.13 ± 4.45 ^A^	20.82 ± 1.52 ^B^	19.23 ± 2.22 ^B^	18.29 ± 1.41 ^B^	<0.01
CO_2_ mL/g DM	179.38 ± 42.49	177.77 ± 11.30	156.52 ± 17.32	169.34 ± 6.65	0.35
F					
TGP mL	142.48 ± 15.22	136.86 ± 2.88	143.55 ± 4.30	138.51 ± 7.16	0.51
CH_4_ mL	12.08 ± 2.27 ^a^	10.00 ± 0.92 ^b^	12.27 ± 0.88 ^a^	11.86 ± 0.98 ^a^	0.03
CO_2_ mL	89.79 ± 21.28	90.75 ± 4.41	96.53 ± 3.71	91.18 ± 5.66	0.73
TGP mL/g DM	284.62 ± 30.35 ^a^	267.97 ± 5.69 ^ab^	273.08 ± 8.11 ^ab^	251.57 ± 12.95 ^b^	0.02
CH_4_ mL/g DM	24.13 ± 4.54 ^a^	19.58 ± 1.81 ^b^	23.34 ± 1.67 ^ab^	21.55 ± 1.79 ^ab^	0.04
CO_2_ mL/g DM	179.38 ± 42.49	177.70 ± 8.73	183.64 ± 7.03	165.61 ± 10.26	0.56

D, dried treatment; F, freeze-dried treatment; CON, control group; 2%, CON plus *S*. *mcclurei* (20 mg/g DM); 5%, CON plus *S. mcclurei* (50 mg/g DM); 10%, CON plus *S. mcclurei* (100 mg/g DM). ^ab^ Means bearing different superscripts in the same row differ significantly (*p* < 0.05). ^AB^ Means bearing different superscripts in the same row differ significantly (*p* < 0.01).

**Table 2:** Chemical composition of substrates used in the in vitro rumen fermentation.

Parameter (% of DM).	Corn Straw	Concentrate ^1^	*S. mcclurei*
DM	26.26	93.53	NA
OM	92.85	92.81	79.17
CP	7.12	20.46	12.71
NDF	38.48	16.61	20.85
ADF	21.69	6.22	16.89
Ash	7.14	7.18	20.82

DM, dry matter; OM, organic matter; CP, crude protein; NDF, neutral detergent fiber; ADF, acid detergent fiber; NA: not available. ^1^ Composition of concentrate: corn 500 g/kg, DDGS (distiller’s dried grains with solubles) 235 g/kg, soybean meal 220 g/kg, stone powder 10 g/kg, dicalcium phosphate 9 g/kg, multivitamin 4 g/kg, salt 10 g/kg, multi-mineral 1 g/kg, baking soda 10 g/kg, mold inhibitor 1.5 g/kg.

**Table 3:** Effect of the two treatments and different supplementation levels *S. mcclurei* on nutrient degradation in vitro.

Parameter	CON	2%	5%	10%	*p*
D					
DMD %	84.78 ± 2.84	84.78 ± 4.13	84.89 ± 2.21	82.61 ± 2.40	0.49
NDFD %	80.45 ± 3.66	80.85 ± 5.20	79.08 ± 3.06	78.90 ± 2.92	0.76
ADFD %	79.22 ± 3.89	78.53 ± 5.83	74.14 ± 3.78	75.44 ± 3.40	0.16
CPD %	90.83 ± 2.21	91.66 ± 2.26	91.30 ± 1.27	89.84 ± 3.27	0.38
F					
DMD %	84.78 ± 2.84	79.22 ± 2.01	83.66 ± 2.59	82.33 ± 2.56	0.33
NDFD %	80.45 ± 3.66	80.30 ± 2.57	82.16 ± 2.82	77.49 ± 3.27	0.10
ADFD %	79.22 ± 3.89	77.28 ± 2.97	75.84 ± 3.82	74.31 ± 3.73	0.14
CPD %	90.83 ± 2.21 ^AB^	93.85 ± 2.09 ^A^	90.85 ± 1.06 ^AB^	87.85 ± 1.51 ^B^	<0.01

D, dried treatment; F, freeze-dried treatment; CON, control group; DMD, degradation of dry matter; NDFD, degradation of neutral detergent fiber; ADFD, degradation of acid detergent fiber; CPD, degradation of crude protein; 2%, CON plus *S. mcclurei* (20 mg/g DM); 5%, CON plus *S. mcclurei* (50 mg/g DM); 10%, CON plus *S. mcclurei* (100 mg/g DM). ^AB^ Means bearing different superscripts in the same row differ significantly (*p* < 0.01).

**Table 4:** Effect of the two treatments and different supplementation levels of *S. mcclurei* on pH, NH_3_-N, MCP, and VFA profiles in vitro.

Parameter	CON	2%	5%	10%	*p*
D					
pH	6.95 ± 0.11	6.95 ± 0.11	6.94 ± 0.05	6.95 ± 0.10	0.99
NH_3_-N mmol/L	14.52 ± 0.84	13.29 ± 2.10	13.92 ± 0.61	14.86 ± 0.84	0.17
MCP μg/mL	44.24 ± 8.88 ^b^	59.16 ± 7.61 ^a^	61.21 ± 9.43 ^a^	62.75 ± 12.84 ^a^	0.01
Total VFA mmol/L	79.39 ± 5.31 ^A^	65.55 ± 5.52 ^B^	65.72 ± 6.42 ^B^	64.34 ± 8.34 ^B^	<0.01
Acetate mmol/L	45.78 ± 2.43 ^A^	37.18 ± 3.30 ^B^	37.24 ± 3.72 ^B^	36.35 ± 4.95 ^B^	<0.01
Propionate mmol/L	19.36 ± 1.93	17.36 ± 1.40	17.48 ± 1.64	17.47 ± 1.96	0.17
Butyrate mmol/L	10.15 ± 0.71 ^A^	7.85 ± 0.68 ^B^	7.87 ± 0.74 ^B^	7.58 ± 1.03 ^B^	<0.01
A:P	2.37 ± 0.10 ^A^	2.14 ± 0.09 ^B^	2.13 ± 0.08 ^B^	2.07 ± 0.06 ^B^	<0.01
F					
pH	6.95 ± 0.11	6.93 ± 0.12	6.93 ± 0.09	6.98 ± 0.11	0.83
NH_3_-N mmol/L	14.52 ± 0.84	14.91 ± 0.28	14.35 ± 1.02	14.33 ± 1.38	0.7
MCP μg/mL	44.24 ± 8.88	51.08 ± 15.82	57.53 ± 5.71	52.19 ± 15.02	0.32
Total VFA mmol/L	79.39 ± 5.31 ^a^	66.43 ± 10.71 ^b^	72.03 ± 7.56 ^ab^	77.20 ± 2.37 ^a^	0.02
Acetate mmol/L	45.78 ± 2.43 ^A^	37.55 ± 6.14 ^B^	41.48 ± 4.57 ^AB^	44.97 ± 1.49 ^A^	<0.01
Propionate mmol/L	19.36 ± 1.93	17.76 ± 2.78	18.04 ± 1.48	18.77 ± 0.65	0.46
Butyrate mmol/L	10.15 ± 0.71 ^A^	7.92 ± 1.26 ^B^	8.90 ± 1.06 ^AB^	9.58 ± 0.36 ^A^	<0.01
A:P	2.37 ± 0.10 ^A^	2.11 ± 0.07 ^B^	2.29 ± 0.10 ^A^	2.39 ± 0.06 ^A^	<0.01

D, dried treatment; F, freeze-dried treatment; CON, control group; 2%, CON plus *S. mcclurei* (20 mg/g DM); 5%, CON plus *S. mcclurei* (50 mg/g DM); 10%, CON plus *S. mcclurei* (100 mg/g DM), A:P, acetate/propionate ratio. ^ab^ Means bearing different superscripts in the same row differ significantly (*p* < 0.05). ^AB^ Means bearing different superscripts in the same row differ significantly (*p* < 0.01).

**Table 5:** Alpha diversity indices of bacteria and archaea among treatments at the 2% supplementation level in vitro.

Parameter	CON	F	D	*p*
Bacteria				
ACE	1508.08 ± 138.74	1507.12 ± 40.80	1431.51 ± 111.04	0.37
Chao 1	1496.73 ± 130.89	1482.26 ± 53.17	1415.56 ± 91.87	0.33
Shannon	5.01 ± 0.24	5.08 ± 0.19	4.91 ± 0.21	0.41
Simpson	0.04 ± 0.01	0.03 ± 0.01	0.04 ± 0.01	0.22
Archaea				
ACE	379.55 ± 139.69	267.21 ± 162.74	318.11 ± 139.8	0.43
Chao 1	378.33 ± 140.38	259.38 ± 166.58	310.96 ± 141.32	0.40
Shannon	1.21 ± 0.17	0.99 ± 0.30	1.07 ± 0.18	0.26
Simpson	0.58 ± 0.04	0.61 ± 0.05	0.63 ± 0.06	0.28

CON, control group; F, freeze-dried treatment (2% of substrate DM); D, dried treatment (2% of substrate DM); ACE, abundance-based coverage estimator.

## Data Availability

All data supporting the present study are reported in this study. Sequence data presented in this study are openly available in the NCBI database.
